# Developmental pathways of repetitive non-suicidal self-injury: predictors in adolescence and psychological outcomes in young adulthood

**DOI:** 10.1186/s13034-023-00660-5

**Published:** 2023-10-13

**Authors:** Margit Wångby-Lundh, Lars-Gunnar Lundh, Benjamin Claréus, Jonas Bjärehed, Daiva Daukantaitė

**Affiliations:** Department of Psychology, Lund University, Lund, Sweden

**Keywords:** Non-suicidal self-injury, Individual developmental pathways, Late onset, Cessation, Depressive symptoms, Poor sleep

## Abstract

**Background:**

Much longitudinal research has been carried out on non-suicidal self-injury (NSSI) during the last decades, but there still is a lack of studies of the individual developmental pathways of NSSI from adolescence into young adulthood. The aim of the present study was to investigate individual developmental pathways of *repetitive non-suicidal self-injury* (repNSSI) from adolescence into young adulthood, including adolescent predictors and psychological outcomes in young adulthood. Three developmental pathways were targeted: *stable adolescence-limited repNSSI*; *repNSSI prolonged into young adulthood*; and *late-onset repNSSI*; with *no repNSSI* as comparison.

**Methods:**

Data were taken from a cohort of compulsory school students (N = 1064) in grades 7–8 in a Swedish municipality. The cohort was followed longitudinally, and this study included all individuals (n = 475) with NSSI data from three waves: T1 (when they were 13–15 years old); T2 (one year later); and T3 (ten years later). RepNSSI was operationalized as self-reports of at least 5 instances of NSSI during the past six/twelve months.

**Results:**

The two pathways that involved stable repNSSI were observed significantly more often than expected by chance, with the strongest overrepresentation for the Prolonged RepNSSI pathway. Still, most adolescents who engaged in stable repNSSI stopped this before reaching young adulthood. Those who stopped did not, however, show a significantly better psychological adjustment in young adulthood than those who continued. Compared to participants with no repNSSI, participants who had stopped still reported significantly more stress, anxiety, and emotional dysregulation. As to the prediction of late onset NSSI, the findings were less robust, but sporadic NSSI at T1 and poor sleep at T2 were significant predictors, whereas depressive symptoms fell just short of significance at both timepoints.

**Conclusions:**

The present results indicate that among adolescents who engage in stable adolescent repNSSI (1) significantly more individuals than expected by chance still engage in repNSSI ten years later, and (2) those who stop engaging in repNSSI do not show significantly better psychological adjustment than those who still engage in it. The present findings also indicate that late onset of repNSSI as reported in young adulthood to some extent is predictable from symptom measures ten years earlier.

## Background

During the last decades many longitudinal studies have been investigating the development of non-suicidal self-injury (NSSI) in adolescence and young adulthood. Many of these studies have had a correlational approach, studying general models of the development of NSSI, associations between risk factors and subsequent development of NSSI as well as between NSSI and subsequent outcomes [e.g., 1–4]. Much less longitudinal research has focused on investigating the different *individual developmental pathways* that NSSI takes, the risk factors related to these developmental pathways and their psychological outcomes. An increased understanding of the developmental course of NSSI at the individual level is essential in the ongoing search for prevention methods and treatments in this area.

It is known that NSSI often takes its starting point in early adolescence, with a peak prevalence between 14 and 16 years, and appears to decrease by the age of 18 years [5] or at least before 21 years of age [6]. Age-related prevalence rates, however, do not tell us anything about how NSSI tends to develop for individuals. How common is it that individuals who engage in NSSI in early adolescence continue to do so as young adults, and how common is it that they stop? How common is it that individuals who do not engage in NSSI in early adolescence start to engage in it at a later stage? Gandhi et al. [7] reported a second peak of NSSI around the age of 20 in a community sample, and around the age of 24 in a psychiatric sample. These findings, however, were based on retrospective data, and the authors suggested that one explanation could be recall bias – that the young adults tended to remember the most recent instances of NSSI and failed to recall earlier episodes that had occurred during their adolescent years. This raises the question to what extent late onset NSSI is a real phenomenon and not just the result of a recall bias.

To answer these types of questions one needs access to prospective, longitudinal data following individuals over the crucial developmental periods, so that each individual may be represented by their specific *developmental pathway*^1^. In the present study, we wanted to investigate the different individual developmental pathways that NSSI takes in a longitudinal general-population sample of teenagers entering young adulthood. We were particularly interested in three subgroups of young people with different types of pathways: individuals with stable NSSI in adolescence *prolonged* into young adulthood; individuals with stable NSSI *only in adolescence*; and individuals with a *late onset* of NSSI in young adulthood.

### Stability in the individual development of NSSI

In a meta-analysis of prospective risk factors for NSSI, Fox and colleagues [8] found that a prior history of NSSI was the strongest predictor of NSSI, with a high odds ratio (just below 6). This finding is in accordance with theoretical models of self-reinforcing properties in NSSI [9, 10]. Nock [9] suggested in his NSSI model that self-injury may be maintained by fulfilling four functions: intrapersonal reinforcement, either negative (an immediate reduction of negative affective states or aversive tension) or positive (e.g., satisfaction from having punished oneself); or interpersonal reinforcement, which also may be negative (decrease or cessation of negative social experiences, such as bullying or fights between parents) or positive (e.g., receiving attention or support from loved ones; see also [11, 12]). Of these four functions, intrapersonal negative reinforcement has received most attention [11, 13–15]. Use of NSSI may lead to an instant relief of anxiety and other states of painful, psychological tension [16–18]. In the absence of alternative affect regulation strategies, this instant relief is suggested to reinforce the engagement in NSSI as a way of handling strong negative emotions. In their new conceptual model of NSSI, Hooley and Franklin [10] argued that the affect regulation function of NSSI plays a minor role in *initiating* the behaviour, but a major role in *maintaining* it. They proposed that the pain offset relief, which follows immediately when the self-injurious behaviour is ended, may be responsible for an improvement in affect and may function as an unconditioned stimulus that becomes associated with NSSI.

If there is a strong tendency towards individual stability in NSSI once it has been initiated, this could theoretically be expected to influence prevalence rates of different individual pathways of NSSI. In the present study, we wanted to investigate how common different individual pathways were in our longitudinal general-population sample, while using configural frequency analysis [19] to detect possible associations between stability and the prevalence rates of different pathways.

### Individual developmental pathways

To our knowledge, there are no prospective, longitudinal studies so far that have investigated the different individual developmental pathways that NSSI takes from adolescence into young adulthood. There are, however, a few prospective, longitudinal studies of individual developmental pathways of NSSI during adolescence [20–22]. These studies were specifically focused on identifying *latent trajectories* underlying/explaining the observed individual data patterns of NSSI over time. Two of these studies were made in China [20, 21]. Barrocas et al. [20] studied NSSI data from a sample of 617 adolescents across a 2-year period, from age 15 to age 17. Through group-based trajectory modelling, they identified three NSSI trajectory classes of NSSI engagement: *low* (69.2% of participants), *moderate* (26.1%) and *chronic* (4.7%). Wang et al. [21] used an accelerated longitudinal design following 3,381 adolescents, aged 13 to 17 years, over a 1-year period. With latent class growth analysis, they identified four distinct NSSI trajectory groups: *negligible* (74.6%), *experimental* (12.8%), *moderate decreasing* (10.8%) and *high fluctuating* (1.9%).

The third of these studies was made among adolescents in Sweden. Tilton-Weaver and colleagues [22] used latent growth curve modelling to analyse cohort-sequential NSSI data from the longitudinal research program Three Cities Study. Data were collected in five annual waves among school students aged 13 and 17. In their analytical sample of 3,195 adolescents, three developmental NSSI trajectories were identified: a *stable-low* trajectory, comprising 94% of the sample; a *low‐increasing* trajectory (3%), which started low but showed an exponential increase during adolescence that did not reach a plateau during the studied age interval; and an *increasing‐decreasing* trajectory (3%), starting at a higher level than the other trajectories and increasing from this level until the age of 15, after which the NSSI levels instead decreased. The authors noted that they did not find any trajectory similar to the trajectory with high and fluctuating NSSI levels identified by Wang et al. [21], which was very low-frequent in that study (1.9%). They concluded that NSSI appears to develop along multiple trajectories and that it is important to identify even very low-frequent trajectories to explain change in self-injury during adolescence.

In the present study, we were interested in extending the investigation of individual developmental pathways of NSSI from adolescence into young adulthood. In contrast to earlier studies of individual pathways, however, we were not searching for underlying latent trajectories. Instead, our aim was to consider all theoretically possible individual NSSI pathways, even if they were low-frequent or not observed at all, as very unusual or non-existent individual pathways also tell us something about the developmental course of NSSI (so-called “white spots” [23]). To do this we used configural frequency analysis [19, 23], which is a method for studying all possible patterns of values on a set of discrete variables, preferably dichotomized. We therefore reduced our continuous measures of NSSI to binary NSSI variables.

There are at least two earlier prospective longitudinal studies which have applied an approach that is similar to ours by studying subgroups of participants who follow different observed individual pathways of NSSI. In a one-year longitudinal study on the association between body objectification and NSSI [24], Duggan and colleagues followed two groups of adolescents (11–13 years of age): one group that desisted from their self-reported NSSI between the first and second data collection (*the NSSI Stop group*); and another group that maintained their self-reported NSSI over the two data collections (*the NSSI Maintenance group*). In another one-year longitudinal study among university students [25], Hamza and Willoughby investigated five subgroups among students reporting NSSI (lifetime history NSSI at Time 1, NSSI in the past year at Time 1, and/or NSSI in the past year at Time 2): *new beginners* (not reporting any history of NSSI at Time 1, but new onset of NSSI in the past year at Time 2); *recovered injurers* (reporting a lifetime history of NSSI at Time 1, although not in the past year, neither at Time 1 nor at Time 2); *relapsers* (not reporting NSSI in the past year at Time 1, but reporting NSSI in the past year at Time 2); *desisters* (reporting NSSI in the past year at Time 1 but not at Time 2); and *persistent injurers* (reporting NSSI in the past year both at Time 1 and at Time 2). Unlike the trajectory studies cited above and our own study, these two studies were limited to two waves of data collection. Moreover, neither of these two studies focused on prevalence rates of individual pathways of NSSI in representative general-population samples.

### Risk factors for different individual developmental pathways

A second set of questions in the present study, besides investigating the individual developmental pathways of NSSI per se, concerns the predictors of the three specific individual developmental pathways in focus of this study: stable NSSI in adolescence *prolonged* into young adulthood; stable NSSI *only in adolescence*; and a *late onset* of NSSI in young adulthood. Among individuals who self-injure in adolescence, how can we tell who has an increased risk of continuing their adolescent NSSI behaviour into adulthood, and who is likely to stop their NSSI behaviour? And among individuals who do not self-injure in adolescence, how can we know who is at risk of a late onset of NSSI? If there are ways of identifying risk factors early in adolescence for different types of long-term developmental pathways of NSSI, we might learn more about how to help different young persons in the best ways.

Considering NSSI in general, Fox et al. [8] concluded that there were few strong predictive NSSI risk factors identified in the literature: Besides prior history of NSSI, only cluster B personality disorders, and hopelessness yielded odds ratios > 3.0. Two other systematic reviews, by Plener et al. [5] and Valencia-Agudo et al. [26], of prospective longitudinal predictor studies pointed to depressive symptoms as one of the most common risk factors for NSSI.

There is also little evidence of predictors that differentiate between specific developmental pathways of NSSI. Barrocas et al. [20], however, found that negative attributional style could distinguish the *chronic* trajectory class both from the *low* and from the *moderate* classes. Also, Tilton-Weaver et al. [22] found some risk factors that differentiated between all three of the trajectories identified in their study. These risk factors were family-related stress at age 13 and at age 16, peer victimization at age 13, and symptoms of depression and anxiety at age 13 and at age 16. Adolescents in the *increasing-decreasing* trajectory class fared worst in all comparisons, followed by the adolescents in the *low-increasing* class.

#### Risk factors for late onset

There are few longitudinal studies of risk factors for late onset NSSI. Most of the studies reviewed by Fox and colleagues [8] had rather short follow-up lengths, ranging from 0.45 to 108 months, with a mean follow-up length of 20.65 months (median = 12 months). Fox and colleagues also pointed out that risk factors for *continued* NSSI and risk factors for *onset* of NSSI may not be the same, and that only two of the 20 studies included in their meta-analysis, specifically investigated risk factors for NSSI onset.

Late onset was studied in a large population-based longitudinal study by Moran et al. [27] with seven waves of measurements. However, they did not differentiate between suicidal and non-suicidal self-harm, and it remains unclear to what extent their results apply specifically to NSSI. Their findings suggested that clinically relevant symptoms of depression and anxiety in adolescence (i.e., between 15 and 17 years of age) were associated with new incidence of self-harm (suicidal or non-suicidal) in young adulthood (i.e., between 20 and 29 years of age).

Kiekens and collegues [28] investigated the new onset of NSSI in a prospective longitudinal study of a large sample of college students in Belgium with measures at two timepoints. Various traumatic experiences before the age of 17 (retrospective data at entering the college) increased the risk of new onset of NSSI in college, but multivariate models suggested that different forms of abusive family relationships increased the risk of college onset of NSSI through re-victimization in peer and partner relationships prior to age 17. Mental health problems were also prospectively associated with new college onset of NSSI, but here multivariate models suggested that role impairment might partially account for these associations.

In our own previous longitudinal studies of NSSI in adolescence where we used the present sample (studying predictors over a one-year period), we found evidence that general psychological difficulties [29], depressive symptoms [30] and poor sleep [31] in early adolescence may predict new incidence of repetitive NSSI one year later. In the present study, we wanted to extend these findings by investigating if these problems in adolescence could be risk factors also for late onset of repetitive NSSI, as reported ten years later. To our knowledge, no previous study has examined predictors of onset of NSSI specifically over such a long age period.

### Cessation of NSSI

Cessation of NSSI has often been studied in qualitative studies among participants with lived experience of NSSI, and is described as a complex process with interacting cognitive, behavioural, and relational aspects (e.g., [32]). Recognition of the harmful consequences of NSSI may enforce a decision to stop injuring oneself [33, 34], and encourage the individual to seek help from others [35]. During this process, individuals who previously self-injured may express ambivalence whether their newly acquired strategies will be helpful in all situations [36] and may be likely to injure themselves again if they feel the need to [37, 38]. If the discontinuation of NSSI is to last, change probably needs to extend beyond the cessation of NSSI [39], and include an improved living situation such that there is a reduction of the contextual antecedents of NSSI [40], or that changes in one’s perceptions of the self, and of the benefits of NSSI, increase the barriers for engagement in NSSI [10].

It is quite possible that distal risk factors play a role in complicating the cessation process. For example, if depression in early adolescence has a negative impact on well-being and social development in adulthood (e.g., [41, 42]) this might make it more difficult to stop engaging in NSSI. In the present study, we wanted to test if distal factors, such as general psychological difficulties, depressive symptoms, and poor sleep are predictors of the prolongation of NSSI into young adulthood.

### Outcome in young adulthood

Our third set of research questions concerns outcome in young adulthood, that is, whether different developmental pathways of NSSI are associated with differences in mental health and well-being in young adulthood. Some research findings indicate long-term detriments to subjective wellbeing even in individuals who have stopped engaging in NSSI [6]. But how do individuals who have stopped their NSSI engagement compare to individuals following other developmental pathways of NSSI? In a previous study based on the same data [43] we found that adolescents who reported stable repetitive NSSI over a one-year interval showed significantly elevated levels of emotional difficulties (stress, anxiety, NSSI, and difficulties in emotion regulation) 10 years later. In that study, however, we did not differentiate between adolescents with stable repetitive NSSI that was *continued* into young adulthood and adolescents whose repetitive NSSI was limited to adolescence. Would the results from our earlier study of elevated levels of young adult emotional problems among participants with stable adolescent repetitive NSSI apply to both these groups? In addition, we were interested in the emotional adjustment situation in young adulthood for individuals with the third type of NSSI development, the late-onset pathway.

### The present study

The overall purpose of this study was to investigate individual developmental pathways of NSSI from adolescence into young adulthood by using data from three time points: T1 (when the participants were 13–15 years old); T2 (one year later); and T3 (ten years later). To make the study as clinically relevant as possible, we chose to highlight the severe end of the NSSI behaviour continuum and focus on the individual development of *repetitive* NSSI (that is, NSSI not just experimented with but performed repeatedly). In accordance with previous research [44, 45], we defined repetitive NSSI (henceforth *repNSSI*) as engagement in NSSI with five or more instances of NSSI reported during the past six months (in young adulthood: 12 months). This definition is also relatively close to the suggested frequency criterion for NSSI disorder in DSM-5 [46]. Our research questions related to individual developmental pathways of NSSI may be summarized as follows:

*First*, we wanted to consider all theoretically possible individual developmental pathways based on presence/absence of repNSSI at the three time points, to see which were observed and how common they were. The prevalence rates of different individual developmental pathways of repNSSI tell us about the absolute risks for stable problems and the chance of remissions. As described above, theoretical models and empirical findings have suggested that NSSI, at least once the behaviour is recurrent, might have a tendency towards maintenance. Generally, this would imply that individual developmental pathways involving stability should be more common than they would be if there were no such tendency towards maintenance in repNSSI. To explore this, we compared the observed frequencies of different pathways with the frequencies expected by chance without the tendency towards maintenance. We also wanted to explore the gender distribution among individuals with different developmental pathways.

*Second*, we were particularly interested in studying the developmental pathways characterized by (a) stable repNSSI that was limited to adolescence and had stopped in young adulthood (labelled *Stable Adolescence-Limited RepNSSI*); (b) stable adolescent repNSSI continuing into young adulthood (*Prolonged RepNSSI*); and (c) repNSSI reported for the first time in young adulthood (*Late-Onset RepNSSI*). We wanted to know if there are risk factors in adolescence that may predict a late onset of repNSSI among individuals not reporting repNSSI in adolescence; and that may predict cessation vs. prolongation into young adulthood among individuals with stable repNSSI in adolescence. *Finally*, we wanted to know what implications different developmental pathways of repNSSI may have on young adult psychological adjustments in general.

Two core concepts here are “repetitive” and “stable”. *Repetitive* refers to repeated occurrences of NSSI during a given period, as measured retrospectively at a single time-point. *Stable*, on the other hand, refers to stability across time-points. Importantly, this operationalization of stability implies *stability in reports over time points*, but it does not imply continuity in behaviour. It may well be, for example, that individuals who showed stability in reports of repNSSI across all three time-points still refrained from engaging in repNSSI during the long period between the second and third time-point when we did not collect any reports.

## Method

The present study had a prospective, longitudinal design and was part of a large research project, which is following a community cohort of compulsory school students in a Swedish middle-sized municipality (around 40,000 inhabitants).

### Participants

The community cohort in this study comprised all compulsory school students in Grade 7 and 8 (N = 1,064) in 2007. Students attending schools for students with learning disabilities were not included, however. Normally, students in Sweden start in the 7th grade the year when they turn 13. The study includes three data collection waves. Of the students in the cohort, 991 (93%; 50.3% girls) participated in the data collection at Time 1 (T1) in 2007. In a new data collection one year later, at Time 2 (T2) 984 (out of 1,098 eligible) students participated (90%; 51.1% girls). The total number of eligible students at T1 and/or T2 was 1,109, which was the target sample for the data collection at the 10-year follow-up (T3) in 2017. Of the individuals in this sample, 557 participated (response rate: 50.2%; 59.2% women). In the present study, we included those participants (n = 475) who had data on NSSI from all three waves of the project. Table 1 presents the descriptive statistics at T3 for this longitudinal sample. Most participants were married/cohabiting or were in a relationship (63.2%) and were part or full-time employees (61.5%) at T3.

**Table 1:** Demographic Characteristics at T3 of the Longitudinal Sample (N = 475)

Variable	Women n = 273	Men n = 202	Total N = 475
**Marital Status**			
Single	87 (31.9%)	88 (43.6%)	175 (36.8%)
Married/Cohabiting with partner	151 (55.3%)	89 (44.1%)	240 (50.5%)
In a relationship	33 (12.1%)	22 (10.9%)	55 (11.6%)
Divorced	0	1 (0.5%)	1 (0.2%)
Other	2 (0.7%)	2 (1.0%)	4 (0.8%)
**Education Level**			
Lower secondary education	5 (1.8%)	5 (2.5%)	10 (2.1%)
Gymnasium	118 (43.2%)	103 (51.0%)	221 (46.5%)
Single university courses	21 (7.7%)	22 (10.9%)	43 (9.1%)
University degree (< 3 years)	21 (7.7%)	13 (6.4%)	34 (7.2%)
University degree (≥ 3 years)	102 (37.4%)	53 (26.2%)	155 (32.6%)
Other	6 (2.2%)	6 (3.0%)	12 (2.5%)
**Working Status**			
Student	70 (25.7%)	54 (26.9%)	124 (26.2%)
Full-time/part-time employed	165 (60.7%)	130 (64.7%)	295 (62.4%)
On sick leave	5 (1.8%)	1 (0.5%)	6 (1.3%)
On parental leave	15 (5.5%)	0	15 (3.2%)
Unemployed	10 (3.7%)	11 (5.5%)	21 (4.4%)
Other	7 (2.6%)	5 (2.5%)	12 (2.5%)

### Measures

#### Non-suicidal self-injury (NSSI)

The Deliberate Self-Harm Inventory, short 9-item version (DSHI-9r) was used to assess self-harm across three waves. DSHI-9r is a shortened and modified version of Gratz’s Deliberate Self-Harm Inventory (DSHI; [47]), adapted to Swedish adolescents [48, 49] and then revised [29]. Respondents were instructed to rate how often they had deliberately engaged in nine different self-harm behaviours (i.e., cutting, minor cutting causing bleeding, burning, punching/banging oneself, biting, carving, severe scratching, sticking sharp objects into skin, and preventing wounds from healing) during the past six (T1 and T2) or twelve (T3) months, on a scale from 0 (“never”) to 6 (“more than five times”). The scores on the nine items are summarized into a total NSSI score. The DSHI-9r shows good test–retest reliability [49]. In the present study, Cronbach’s alpha for DSHI-9r were 0.90 (T1), 0.89 (T2) and 0.81 (T3).

##### RepNSSI

Repetitive NSSI was defined as reports of at least 5 instances of self-harm during the past six (T1 and T2) or twelve (T3) months. A dichotomous measure of presence or not of repetitive NSSI was labeled *repNSSI* and computed from the total NSSI score. Total scores of 0 to 4 rendered a value of 0 for repNSSI, and total scores of 5 or higher rendered a value of 1. Please note that a total score of 5, for example, could be achieved in different ways: by reporting one instance of self-harm with each of five different methods, by reporting five instances of self-harm with one and the same method, or with some other combinations.

##### Individual developmental pathways of repNSSI

The individual developmental pathways were represented by three-digit value patterns formed by the individuals’ values in the measure of repNSSI at the three different time points. The pathway of an individual not reporting repNSSI at any of the three time points was represented by the value pattern 000; an individual reporting repNSSI only at the first time point was represented by the value pattern 100; and so on. With dichotomous measures of repNSSI at three time points there were eight (2 × 2 × 2) theoretically possible value patterns. All eight value patterns and their observed frequencies in the longitudinal sample are presented in Table 2, together with the mean values (and *SDs*) on total NSSI at T1, T2, and T3 for the eight groups with the different value patterns.

**Table 2:** Participant Categorization According to Individual Longitudinal Value Patterns (N = 475): (a) NSSI Total at T1, T2 and T3 (Mean and SD); (b) Configural Frequency Analysis (Observed and Expected Frequencies); and (c) Gender Distribution for Each Value Pattern

Pattern Labels	NSSI Total				Value Pattern				Configural Frequency Analysis						Gender	
	T1	T2	T3		T1	T2	T3		Observed	Expected	*χ* ^*2*^	*p*	Type/ Antitype		Women	Men
**No RepNSSI**	0.43 (0.88)	0.44 (0.95)	0.12 (0.51)		0	0	0		328	282.27	7.41	< 0.0001	Type		173^ A^	155^T^
**Late-Onset RepNSSI**	1.00 (1.41)	0.82 (1.26)	11.50 (10.80)		0	0	1		22	31.00	2.61	0.104			12	10
RepNSSI only at T2	0.94 (1.24)	13.64 (13.02)	0.30 (0.81)		0	1	0		47	78.15	12.42	< 0.0001	Antitype		29	18
RepNSSI at T2 and T3	0.00 (0.00)	13.00 (3.61)	12.67 (10.69)		0	1	1		3	8.58	3.63	0.055			2	1
RepNSSI only at T1	12.53 (12.08)	1.26 (1.33)	0.32 (0.75)		1	0	0		19	52.92	21.75	< 0.0001	Antitype		12	7
RepNSSI at T1 and T3	8.00 (2.65)	2.00 (2.00)	9.00 (5.20)		1	0	1		3	5.81	1.36	0.334			2	1
**Stable Adol.-Lim. RepNSSI**	22.03 (15.21)	16.71 (13.03)	0.44 (0.93)		1	1	0		34	14.65	25.54	< 0.0001	Type		29^T^	5^ A^
**Prolonged RepNSSI**	19.58 (14.89)	22.11 (13.54)	12.47 (8.20)		1	1	1		19	1.61	187.94	< 0.0001	Type		14	5

*Note*. 0 = no repNSSI, 1 = repNSSI. RepNSSI = Repetitive Non-Suicidal Self-Injury (≥ 5 episodes). The labels of the four targeted developmental patterns are in bold

*Configural frequency analysis*: The expected frequencies were based on an independence model after accounting for marginal probabilities. The probabilities of the observed frequencies are given by the binomial distribution. Type = significantly more observations than expected by chance; antitype = significantly less observations than expected by chance; two-tailed tests, with a 5%-significance level.

*Gender distribution*: ^T^ = more common than expected by chance, ^A^ = less common than expected by chance; exact single-cell test based on the hypergeometric distribution, two-tailed tests with a 1%-significance level.

#### Predictors measured at T1 and T2

##### Psychological difficulties

Participants completed the SDQ-s [50] at T1 and T2. SDQ is a widely used screening instrument for psychological difficulties among children and adolescents and contains five subscales with five items each. Four of these measure difficulties: emotional symptoms, hyperactivity-inattention, conduct problems, and peer problems; and the fifth subscale measures prosocial behaviour. Each item is rated on a 3-point scale (0 = true, 1 = somewhat true, and 2 = certainly true) with a time frame of the last 6 months. Five items on the difficulties scales are worded positively and reversed before scoring. In the present study we only used the Total Difficulties score which is the sum for the four difficulties scales. The SDQ was translated into Swedish by Smedje et al. [51], and the self-report version was empirically validated by Lundh et al. [52], who reported a test-retest reliability of 0.72. In the present study, the internal consistency of the total difficulties scale was α = 0.76 and α = 0.75 at T1 and T2, respectively.

##### Depressive symptoms

A Depression Index (DI) was constructed [30] by selecting depression-relevant items from the 11-page questionnaire used at T1 and T2, according to their correspondence with the DSM-IV criteria for major depression [53]. Because the items came from different instruments with different response formats, the scores on each item were transformed to z-scores, before computing the total DI score which was used in the present study. Items referring to positive feelings were reverse scored. The total DI included all items from six subscales: *Dysphoric Relations to Parents* (10 items); *Negative Self-Image* (6 items); *Dysphoric Relations to Friends* (6 items); *Fatigue/Somatic Complaints* (5 items, including a question about poor sleep, see below); *Sadness/Loneliness* (4 items); and *Difficulties in Concentration* (4 items). Test-retest correlations between Time 1 and Time 2 were *r* = .71 for the total DI. In the present study, Cronbach’s alpha for the DI was 0.91 at both T1 and T2.

##### Poor sleep

Poor sleep was assessed by means of one single question, “Do you sleep well?”, with a Likert response format and five response alternatives: 1 = *always*, 2 = *most often*, 3 = *sometimes*, 4 = *seldom*, and 5 = *never*. A pilot study with 80 adolescents who answered this question on two occasions, with a mean test interval of 7 weeks and 4 days, showed a test-retest correlation of *r* = .64 [31]. Because the periods between assessments were longer than is usual in studies of test-retest reliability (which should ideally not be more than about 1 month) the test–retest coefficient obtained was assumed to set a lower boundary for the true test-retest reliability of this measure.

#### Psychological outcomes measured at T3

To investigate the psychological adjustment in young adulthood among groups of individuals following different developmental pathways, we used a set of measures of both positive and negative adjustment at T3.

##### Life satisfaction

The *Satisfaction with Life Scale* (SWLS; [54]) contains 5 items (e.g., “I am satisfied with life”). Participants indicate how much they agree or disagree with each of the 5 items using a 7-point scale that ranges from 1 (*Strongly disagree*) to 7 (*Strongly agree*). Cronbach’s alpha for the scale was 0.92.

##### Flourishing

The *Flourishing Scale* (FS; [55]) is a brief 8-item summary measure of the respondent’s self-perceived success in important areas such as relationships, self-esteem, purpose, and optimism. Participants indicate how much they agree or disagree with each of the 8 items (e.g., “I lead a purposeful and meaningful life”) using a 7-point scale that ranges from 1 (*Strongly disagree*) to 7 (*Strongly agree*). The possible range of scores is from 8 (lowest possible) to 56 (highest possible). A high score represents a person with many psychological resources and strengths. Cronbach’s alpha for the scale was 0.88.

##### Resilience

Resilience was assessed with the *Brief Resilience Scale* (BRS; [56]) that assesses one’s ability to bounce back or recover from stress (e.g., “I tend to bounce back quickly after hard times”). Participants indicate how much they agree or disagree with each of the five items using a 5-point scale that ranges from 1 (*Strongly disagree*) to 5 (*Strongly agree*). The total score is calculated by averaging the item scores. Windle et al. [57] showed that the BRS has sound psychometric properties that are on par with longer measures of resilience. Cronbach’s alpha for the scale was 0.81.

##### Stress, anxiety and depression

The *Depression, Anxiety and Stress Scale* (DASS-21; [58]) was used to assess depression (7 items; e.g., “I felt downhearted and blue”), anxiety (7 items; e.g., “I felt I was close to panic”) and tension/stress (7 items; e.g., “I found it hard to wind down”). Participants responded to each item on a 4-point scale that ranges from 0 (*never*) to 3 (*almost always*). Cronbach’s alphas for depression, anxiety, and stress were 0.90, 0.79, and 0.87, respectively.

##### Emotion dysregulation

The *Brief Difficulties in Emotion Regulation Scale* (DERS-16; [59]) was used to assess participants’ difficulties to regulate emotions, from several aspects including lack of emotional clarity (e.g., “I have difficulty making sense out of my feelings”), difficulties engaging in goal-directed behaviors (e.g., “When I am upset, I have difficulty getting work done”) and controlling impulses (e.g., “When I am upset, I become out of control”), ineffective emotion regulation strategies (e.g., “When I am upset, I believe that I will remain that way for a long time”), and non-acceptance of emotional responses (e.g., “When I am upset, I feel ashamed with myself for feeling that way”). Participants estimated how often each of the 16 statements applied to them using a 5-point scale, ranging from 1 (*almost never*) to 5 (*almost always*), setting the total score at a minimum of 16 and a maximum of 80. The Cronbach’s alpha for DERS-16 was 0.95.

### Procedure

Data collection at T1 and T2 was conducted in collaboration with the municipal body of the selected area and each of the regular schools therein. The headmaster of each school was contacted and agreed to their school’s participation in the study. Data were collected in school settings. Teachers were present but did not take part in the administration, which was conducted by research assistants from Lund University. The students were told that they could feel free to refrain from participation, and that they should not write their names anywhere on the questionnaire to ensure confidentiality. To match the data files from T1 to T2 a pseudo-anonymization procedure was used, which meant that a numeric code was used throughout the research project to designate the identity of the participant on all study documents. The code key was preserved separately from other documents and data files, in a secure place.

To conduct the follow-up at T3, participants’ names from the code lists from the two prior surveys (in accordance with the ethical approval to save the name list of participants for 10 years) were sent to the Swedish state’s personal address register (SPAR) to identify their present locations. After we had received current personal addresses of the participants, letters describing the purpose and procedure of the follow-up were sent to all eligible participants. The eligible participants could complete either a confidential web-survey designed using the Lund University survey system, Survey & Report, or a paper-and-pencil questionnaire. After completion of the survey, participants received two cinema tickets or four lottery tickets as compensation.

### Attrition analyses

#### Attrition between T1/T2 and T3

Attrition analyses were conducted comparing the responders (n = 541) and non-responders (n = 529) at T3 in terms of all variables measured at T1 and T2 [60]. We found some significant differences ranging from very small to small in effect size (Cohen’s *d*/Cramer’s *V* = 0.02‒0.21) but could not identify any clear patterns that differentiated the responders from the non-responders on the variables observed at T1 or T2.

Regarding the variables relevant for the current study, significantly more women than men responded to the survey at T3 (T1 & T2: 51%, T3: 58.4%; *χ*^2^(1) = 29.30, *p* < .001). No significant differences between the responders and non-responders were found on SDQ total, the depression index, sleep problems or NSSI, neither at T1 nor at T2. At T1, the NSSI means±SDs for the longitudinal sample (N = 475) and the T3 non-responders were, respectively, 3.63±8.92 and 3.04±6.30, *t*(910.1) = 1.19, *p* = .235. At T2, the corresponding means±SDs were 3.77±8.50 and 3.33±7.98, *t*(944) = 0.82, *p* = .412. Due to the low number of T1/T2 variables reliably associated with attrition and thus viable as predictor/auxiliary variables in multiple imputation [61], non-responders at T3 were excluded from analysis in the current study.

#### Internal missingness

At each wave, participants having no more than three missing values on the DSHI-9r were included for data analysis. Missing values were interpreted conservatively as absence of the self-injurious behaviour asked for (i.e., imputing 0).^2^ With these imputations we had available data on DSHI-9r for 983 participants at T1, 979 at T2, and 556 at T3. In total, 896 participants had data on DSHI-9r at both T1 and T2, and 475 had data on DSHI-9r at all three waves. These 475 participants comprised the analytic longitudinal sample for this study.

The percentages of participants with internal attrition ranged from 1.3 to 3.2% in the T1 predictor variables, 1.5‒2.9% in the T2 predictor variables, and 1.1‒3.6% in the T3 outcome variables. Little’s [62] Missing Completely At Random (MCAR) test was non-significant (*χ*^2^[874] = 815.77, *p* = .921), suggesting that the internal attrition was MCAR, thereby justifying the inclusion of participants with missing data in the analyses after imputation [63]. Missing data in the different variables were imputed with the Expectation-Maximization algorithm in IBM SPSS.

### Statistical analyses

#### Configural frequency analysis

*A First-Order Configural Frequency Analysis* (CFA; [19]) was used to explore whether the observed frequencies of different individual developmental value patterns were significantly *higher* – or significantly *lower* – than expected by chance in a comparison model. The phenomenon that we wanted to investigate with this analysis was the self-reinforcing property of NSSI, or at least of repNSSI, which should manifest itself in a tendency towards stability of repNSSI.

If NSSI to some extent is self-reinforcing, the engagement in NSSI at one time point would increase the risk of engaging in NSSI also at a subsequent time point. Engaging in NSSI would increase the risk of doing it again. This would mean that presence or absence of repNSSI at the later time point is not totally independent of whether repNSSI was present or not at the previous time point; on the contrary, there would be an *association* between the time points as concerns presence or not of repNSSI. In general, this type of association over time should increase the frequencies of *stable* repNSSI pathways. With three time points, however, some theoretical pathways would involve both stability and change, and we had no theoretical expectations or earlier findings on which we could set up specific hypotheses for these patterns. Therefore, the analyses we made were exploratory.

To analyse whether there was an association between presence/absence of repNSSI over time that influenced the observed frequencies of some of the value patterns, we used a comparison model of *independence* between time points. Based on the independence model, we could compute what frequencies to expect by chance for the various value patterns if the time points were totally independent of each other. Making the computations of expected frequencies, we used the observed *marginal* frequencies, that is, the observed frequencies of presence and absence of repNSSI at each separate time point. RepNSSI was more than twice as common at T2 (21.7% of the longitudinal sample) than at T3 (9.9%), for example, and such differences in marginal frequencies were included in the comparison model.

For each theoretically possible value pattern, the significance of the difference between observed and expected frequencies was tested with a two-tailed test, according to the binomial distribution. For this we used the computer program ROPstat [64]. Patterns that are significantly more common than expected are called *types*, and patterns that are significantly less common are called *anti-types*. It should be noted that whether a pattern is a type or whether it is an antitype is not related to its observed frequency per se. A pattern that is very frequent may be an antitype, and a very low-frequent pattern may be a type. A more elaborate presentation of the CFA analysis is made in Appendix A.

#### Gender distribution among developmental pathways

The gender distribution among developmental pathways was explored with exact single-cell tests, based on the hypergeometric distribution, of each cell in an 8 × 2 cross-tabulation between developmental pathway groups and gender [64, 65].

#### Logistic regression analysis

Logistic regression analyses (fulfilling the assumptions of log-odds linearity, and non-multicollinearity) were conducted to predict membership in (a) the *Late Onset RepNSSI vs. No RepNSSI groups* and (b) the *Stable Adolescence-Limited RepNSSI vs. Prolonged RepNSSI groups*. The regression analyses were performed separately for predictors measured at T1 and predictors measured at T2. The odds ratio (*OR*) was used as an index of effect size. The size of the *OR* was interpreted according to Chen et al. [66], who calculated odds ratio equivalents to Cohen’s *d* and suggested that *OR* = 1.68 should be considered as a small effect size (corresponding to *d* = 0.2), *OR* = 3.47 as a medium effect size, and *OR* = 6.71 as a large effect size.

#### Non-parametric tests of group differences at T3

We also wanted to compare young adult adjustment among the groups following the late onset, cessation, and prolongation pathways as well as the group with stable absence of repNSSI. These groups had unequal sample sizes and non-homogeneous variances, however. Therefore, we used the non-parametric Kruskal-Wallis’ test for the overall comparison of the groups on different psychological outcomes in young adulthood. *Post hoc* tests were performed with the Games-Howell test, which is a non-parametric test that does not assume homogeneity of variances or equal sample sizes. Effect sizes were estimated with Glass’s *delta* using the sample standard deviation of the comparison group. All analyses were carried out using IBM SPSS, version 27.

## Results

### Individual developmental pathways of repNSSI

With the dichotomous measure of repNSSI during the past 6 months at T1 and T2 and the past twelve months at T3, there were eight theoretically possible developmental pathways. As can be seen in Table 2, each of these pathways were observed in the sample, but two of them were very low-frequent (*n* = 3 for each pathway, < 1%). The table also presents the means (and *SD*s) of the NSSI total score at the three time-points for each pathway group.

The analysis we made was exploratory, with two-tailed significance tests (see the Method section and Appendix A). For five developmental value patterns the observed frequencies differed significantly from the frequencies expected by chance based on the independence model. Three of these value patterns were *types*, that is, they were observed significantly more often than expected by chance. These patterns represented the following pathways: *No repNSSI*, *Stable Adolescence-Limited RepNSSI* and *Prolonged RepNSSI*. The strongest type was *Prolonged RepNSSI*, which was observed almost 12 times more often than expected by chance with the independence model. This pattern reflected stability in repNSSI through adolescence into young adulthood. Nineteen participants (4% of the longitudinal sample) reported this pattern while only 1.6 were expected (*p* < .0001, two-tailed test). The second type pattern, *Stable Adolescence-Limited RepNSSI*, was observed for 34 participants (7.2%), which was more than twice as many as expected by chance (exp.: 14.6; *p* < .0001, two-tailed test). This pattern reflected stability in repNSSI during adolescence, but a cessation in young adulthood. The third significant pattern, *No RepNSSI* was not a very strong type, observed only 1.2 times more often than expected (obs.: 328, exp.: 282.3; *p* < .0001, two-tailed test). This pattern reflected absence of repNSSI at each time point.

Two value patterns came out of the analysis as significant *antitypes*, that is, they were significantly less common than expected by chance. These patterns reflected a lack of stability in repNSSI: *RepNSSI only at T1* (4.0%; obs.: 19; exp.: 52.9; *p* < .0001, two-tailed) and *RepNSSI only at T2* (9.8%; obs.: 47; exp.: 78.2; *p* < .0001, two-tailed). In addition, it may be noted that repNSSI limited to one time-point in adolescence was more than twice as common at T2 as at T1. The observed frequency of *Late-Onset RepNSSI* was somewhat lower than expected by chance, but this discrepancy was not significant (4.6%; obs.: 22; exp.: 31.0; *p* = .104).

As is also presented in Table 2, we found significant gender differences in the frequencies of two value patterns. The pattern representing *Stable Adolescent-Limited RepNSSI* was found for 29 women, which was significantly *more* than expected by chance (expected frequency: 19.5; exact single-cell test based on the hypergeometric distribution, *p* < .01). Consequently, the number of men who had this pattern was significantly lower than expected (obs.: 5; exp.: 14.5; exact single-cell test based on the hypergeometric distribution, *p* < .01). The *No RepNSSI* value pattern, on the other hand, was significantly *less* common among women (obs.: 173; exp.: 188.5), and significantly more common among men (obs.: 155; exp.: 139.5; *p* < .01). In addition, the *Prolonged RepNSSI* value pattern was more common among women (observed: 14; exp.: 10.9) than among men (obs.: 5; exp.: 8.1), but the discrepancies were not significant.

### Adolescent predictors of developmental patterns of repNSSI

Three different adolescent risk factors, which in earlier studies of the cohort predicted a new incidence of repNSSI over one year in adolescence, were tested (1) as predictors of a late onset (that is, a new incidence of repNSSI in young adulthood); and (2) as predictors of the prolongation vs. cessation in young adulthood of stable adolescent repNSSI. These risk factors, measured both at T1 and at T2, were psychological difficulties in general (as measured by the SDQ Total Difficulties score), depressive symptoms (Depression Index) and poor sleep. Because there were small differences on the total NSSI scores both at T1 and T2 between the late onset repNSSI group and the no repNSSI group (see Table 2), we also entered NSSI total at T1 or T2 (depending on when the other predictors were measured) as a predictor at step 2 in each of the logistic regression analyses. In this way we both controlled for differences in NSSI total and investigated whether NSSI total was a significant predictor per se.

Because of overlap between the predictors (the question about poor sleep and five items from the SDQ were included among the 35 items in the Depression Index), all regression analyses with the Depression Index as independent variable (which are presented in Table 3) were carried out separately from the regression analyses with SDQ and poor sleep as independent variables (presented in Table 4).

**Table 3:** Separate Logistic Regressions, Predicting RepNSSI Pattern Group Membership from Depressive Symptoms and NSSI (total score on DSHI-9r) at T1 and at T2

Predictors	Late-Onset vs. No RepNSSI						Stable Adolescence-Limited vs. Prolonged				
	B (SE)	Wald	*p*	OR	95% CI		B (SE)	Wald	*p*	OR	95% CI
***T1 Predictor***											
*Step 1*											
Depressive symptoms	1.30 (0.55)	5.47	0.019	3.67	1.24–10.86		0.61 (0.57)	1.14	0.286	1.84	0.60–5.65
Constant	-2.75 (0.25)	123.12	< 0.001	0.06			0.12 (0.42)	0.08	0.774	1.13	
*Step 2*											
Depressive symptoms	1.11 (0.59)	3.49	0.062	3.03	0.95–9.68		0.49 (0.72)	0.47	0.492	1.64	0.40–6.67
NSSI total	0.50 (0.19)	7.32	0.007	1.66	1.15–2.38		0.01 (0.03)	0.07	0.787	1.01	0.96–1.06
Constant	-3.13 (0.32)	98.02	< 0.001	0.04			0.04 (0.52)	0.01	0.942	1.04	
***T2 Predictor***											
*Step 1*											
Depressive symptoms	1.09 (0.53)	4.31	0.038	2.98	1.06–8.34		0.01 (0.51)	0.00	0.982	1.01	0.37–2.73
Constant	-2.64 (0.23)	128.45	< 0.001	0.07			0.53 (0.40)	1.79	0.181	1.70	
*Step 2*											
Depressive symptoms	0.99 (0.55)	3.30	0.069	2.69	0.92–7.83		0.76 (0.71)	1.14	0.285	2.14	0.53–8.64
NSSI total	0.22 (0.19)	1.45	0.229	1.25	0.87–1.80		− 0.0.05 (0.03)	2.45	0.117	0.95	0.90–1.01
Constant	-2.80 (0.28)	102.16	< 0.001	0.06			1.12 (0.56)	4.00	0.046	3.07	

**Table 4:** Separate Logistic Regressions, Predicting RepNSSI Pattern Group Membership from Psychological Difficulties, Poor Sleep and NSSI (total score on DSHI-9r) at T1 and at T2

Predictors	Late-Onset vs. No RepNSSI						Stable Adolescence-Limited vs. Prolonged				
	B (SE)	Wald	*p*	OR	95% CI		B (SE)	Wald	*p*	OR	95% CI
***T1 Predictors***											
*Step 1*											
SDQ total difficulties	0.08 (0.05)	2.05	0.152	1.08	0.97–1.20		0.08 (0.06)	1.93	0.164	1.09	0.97–1.22
Poor sleep	0.25 (0.30)	0.68	0.409	1.28	0.71‒2.32		-0.37 (0.35)	1.12	0.290	0.69	0.35‒1.37
Constant	-3.97 (0.73)	29.38	< 0.001	0.02			0.23 (1.04)	0.05	0.827	1.26	
*Step 2*											
SDQ total difficulties	0.07 (0.05)	1.42	0.233	1.07	0.96–1.19		0.09 (0.07)	1.45	0.228	1.09	0.95–1.26
Poor sleep	0.18 (0.32)	0.34	0.562	1.20	0.65-2-23		-0.37 (0.35)	1.13	0.288	0.69	0.35–1.37
NSSI, total	0.35 (0.20)	2.93	0.087	1.41	0.95-2-10		-0.00 (0.03)	0.01	0.923	1.00	0.95–1.05
Constant	-3.96 (0.75)	28.07	< 0.001	0.02			0.23 (1.04)	0.05	0.827	1.26	
***T2 Predictors***											
*Step 1*											
SDQ total difficulties	0.04 (0.05)	0.51	0.475	1.04	0.94–1.15		-0.01 (0.06)	0.01	0.910	0.99	0.89–1.11
Poor sleep	0.65 (0.26)	6.35	0.012	1.91	1.16‒3.16		0.21 (0.31)	0.45	0.505	1.23	0.67‒2.27
Constant	-4.37 (0.70)	39.27	< 0.001	0.01			0.05 (0.95)	0.00	0.958	1.05	
*Step 2*											
SDQ total difficulties	0.03 (0.05)	0.23	0.634	1.03	0.93–1.13		0.03 (0.06)	0.28	0.596	1.03	0.92–1.17
Poor sleep	0.64 (0.26)	6.06	0.014	1.90	1.14–3.16		0.39 (0.34)	1.32	0.251	1.47	0.76–2.85
NSSI, total	0.21 (0.19)	1.19	0.276	1.23	0.85–1.80		-0.05 (0.03)	3.43	0.064	0.95	0.90-1.00
Constant	-4.37 (0.70)	38.87	< 0.001	0.01			-0.09 (0.97)	0.01	0.926	0.92	

The results of the logistic regression analyses with the Depression Index at T1 and T2 as predictors are summarized in Table 3. The results showed that depressive symptoms both at T1 and at T2 appeared as significant predictors of late repNSSI onset at step 1. The effects, however, fell just short of significance (*p* = .062 at T1 and *p* = .069 at T2) when NSSI total at T1 and T2 was entered at step 2. NSSI total turned up as a significant predictor at T1 but not at T2. Only the model at T1 was significant, *χ*^2^(2) = 11.76, *p* = .003, Nagelkerke *R*^2^ = 0.103. At T2, the model was not significant, *χ*^2^(2) = 5.47, *p* = .065, Nagelkerke *R*^2^ = 0.045.

The results of the logistic regression analyses with SDQ total difficulties and poor sleep at T1 and T2 as predictors are shown in Table 4. As seen in the table, general psychological difficulties in adolescence, as measured with the SDQ-s total difficulties score, did not predict the onset of repNSSI in young adulthood, but poor sleep did so at T2, *p* = .014, even when NSSI total score at T2 was entered at the second step. The model was significant at T2, explaining 7.2% of the variance, *χ*^2^(3) = 9.07, *p* = .028, Nagelkerke *R*^2^ = 0.072, but not at T1, *χ*^2^(3) = 6.02, *p* = .111, Nagelkerke *R*^2^ = 0.051.

We also used logistic regression analyses to examine whether measures of depressive symptoms (Table 3), and measures of psychological difficulties and poor sleep (Table 4) could differentiate the *Stable Adolescence-Limited* and *Prolonged RepNSSI groups*. Even in these analyses, NSSI total at T1 and T2, respectively, was entered at step 2. The results showed that no one of the studied adolescent risk factors significantly differentiated the *Stable Adolescent-Limited* and *Prolonged RepNSSI pathway groups*, neither at T1 nor at T2.

### Young adult psychological outcomes of different developmental repNSSI pathways

Several positive and negative aspects of the psychological adjustment in young adulthood among participants with different developmental histories of repNSSI were considered: life satisfaction, flourishing, resilience, and psychological problems (stress, anxiety, depression, and emotion dysregulation). The young adulthood adjustment in the *Late-Onset*, the *Stable Adolescence-Limited*, the *Prolonged* and the *No RepNSSI* pathway groups was compared, and the results are presented in Table 5.

**Table 5:** Comparisons of Psychological Outcomes in Young Adulthood Between Four Repetitive Self-harm Pattern Groups: No RepNSSI, Stable Adolescence-Limited, Prolonged and Late-Onset. Kruskal-Wallis and Games-Howell Post Hoc Tests

	RepNSSI Pattern Group								Kruskal-Wallis H
Young Adulthood Psychological Outcome	No RepNSSI		Stable Adolescence-Limited		Prolonged		Late-Onset		
	*M*	*SD*	*M*	*SD*	*M*	*SD*	*M*	*SD*	
Life Satisfaction	24.70 ^a^	6.89	23.03	7.38	20.21	9.33	18.76^a^	7.71	16.23***
Flourishing	47.42 ^a^	6.96	45.38	7.04	41.74	9.75	39.68^a^	9.95	22.30***
Resilience	3.50 ^a^	0.79	3.24	0.69	3.17	0.96	2.82^a^	0.82	17.61***
Stress	5.59 ^a,c^	4.34	9.06 ^c^	5.07	9.79	6.69	10.09^a^	5.36	33.66***
Anxiety	2.45 ^a,c^	2.89	4.82 ^c^	4.00	4.95	4.82	7.18^a^	5.95	32.28***
Depression	3.07 ^a^	4.07	4.94	4.70	5.85	5.95	8.00^a^	5.89	28.25***
Emotion Dysregulation	30.50 ^a,b,c^	12.64	41.94 ^c^	14.04	46.73 ^b^	16.69	44.14^a^	16.63	46.76***

*Note.* Means sharing the same superscript are significantly different from each other

RepNSSI = repetitive non-suicidal self-injury (≥ 5 episodes); ***p < .001. No = No RepNSSI; Adol.-Limited = Stable Adolescence-Limited RepNSSI; Prolonged = Prolonged RepNSSI; Late-Onset = Late-Onset RepNSSI.

First, it may be noted that the *rank order of mean values* of the four groups was almost consistent through almost all variables: The *Late-Onset* group showed the largest impairment (i.e., the lowest mean values in measures of positive adjustment, the highest mean values in measures of negative adjustment) in all variables except for Emotion Dysregulation, in which they showed the second largest impairment. The *Prolonged RepNSSI* group showed the second largest impairment in all variables except for Emotion Dysregulation, in which they showed the largest impairment. The *Stable Adolescence-Limited* group consistently showed the third largest impairment in all variables, and as expected, the *No RepNSSI* group consistently showed the best adjustment of the four groups.

The overall comparisons between the four groups were significant for each measure of young adult adjustment. *Post hoc* analyses showed that all differences between the *Late-Onset* group and the *No RepNSSI* group were significant, and that the effect sizes were large. The participants in the *Late-Onset* group reported significantly lower levels of life-satisfaction, flourishing and resilience (Glass’s *delta* = -0.86 to -1.11), and significantly higher levels of stress, anxiety, depression, and emotion dysregulation (Glass’s *delta* = 1.04 to 1.64). The *Stable Adolescence-Limited* group was found to differ from the *No RepNSSI* group in negative measures of adjustment in young adulthood, reporting significantly higher levels of stress, anxiety, and emotion dysregulation (Glass’s *delta* = 0.79–0.93); the differences on the positive measures were small to moderate and non-significant. The only significant difference identified *post hoc* between the *Prolonged RepNSSI* group and the *No RepNSSI* group however, concerned emotion dysregulation (Glass’s *delta* = 1.32) for which the former reported a significantly higher level. Other differences, although displaying mostly large effect sizes (Glass’s *delta* = -0.66 and − 0.81 for Life Satisfaction and Flourishing; 0.96, 0.87, and 0.74 for Stress, Anxiety, and Depression, respectively), did not attain statistical significance. No significant pairwise differences were found *post hoc* among the three NSSI pathways groups.

## Discussion

The purpose of this study was to investigate individual developmental pathways of repetitive NSSI (repNSSI) from adolescence into young adulthood, and potential adolescent predictors and young adult outcomes of these pathways. First, we considered how common different developmental pathways were, and we also explored if the prevalence rates of different pathways appeared to be influenced by the assumed tendency towards maintenance of repNSSI. Next, we focused on predicting three individual pathways of special relevance to our research questions: *cessation* before young adulthood *vs. prolongation* of stable adolescent repNSSI, and *late onset* of repNSSI in young adulthood. Finally, we explored the psychological adjustment in young adulthood of participants following these three developmental pathways.

### Are stable repNSSI pathways more common than expected?

The mechanisms of NSSI maintenance, which were suggested for example in the model by Nock [9], led us to assume that stable individual pathways would tend to occur *more* often than they would without these mechanisms of maintenance. We explored this through analysing the observed frequencies of all conceivable individual developmental pathway of reporting repNSSI or not at three different age levels. We found that the *prolonged stability* pathway, followed by the participants who reported stable repNSSI both in adolescence and in adulthood, was observed almost 12 times more often than would be expected by chance from an independence model. This finding gives a strong support for a tendency towards stability in repNSSI, even over the long developmental period when the adolescents become young adults. Importantly, however, the tendency towards stability in repNSSI did not preclude change to occur for most adolescents with stable repNSSI. Despite the strong tendency towards stability in repNSSI, adolescents with stable repNSSI were much more likely to cease their engagement in repNSSI after the adolescent years than to continue into young adulthood (7.2% vs. 4.0% in the longitudinal sample). The developmental pathway of cessation of stable adolescent repNSSI was also more common than expected by chance, although only twice as common.

Still, our data suggested that it was considerably more difficult to stop engaging in repNSSI when it had gained stability in adolescence than when it had not. Of the participants with full longitudinal data in the present study, 53 individuals showed stable repNSSI in adolescence; of these, 19 individuals (i.e., 35.8%) still engaged in repNSSI as young adults, whereas 34 individuals showed a cessation (i.e., 64.2%). When this is compared with the outcome for those participants who showed repNSSI *only at one* of the measurement points in adolescence the perspective changes. Seventy-two participants showed this kind of non-stable repNSSI in the present study; of these, only six individuals (i.e., 8.3%) still engaged in repNSSI as young adults, whereas 66 had stopped engaging in repNSSI (i.e., 91.7%). In summary, two thirds of the participants with a stable adolescent repNSSI did not continue to harm themselves repeatedly in young adulthood, which means that change was more common than not among these participants. Yet, the one-year stability in adolescence did involve a substantially increased risk of repNSSI in young adulthood as compared to the risk associated with repNSSI without stability in adolescence.

That so many adolescents with stable repNSSI had ceased their engagement in repNSSI may suggest some kind of spontaneous process. General developmental processes during adolescence and young adulthood may promote changes even in established problematic behaviours. One such process is the maturation of the brain. During adolescence, there are large structural and functional changes to brain regions implicated in the generation of emotions and in the regulation of emotions [67]. Several researchers have argued that emotional control mechanisms are underdeveloped relative to their emotion generating counterpart in adolescence. This would to some extent account for the observed deficiencies in emotion regulation during adolescence and the adolescent affective and behavioural problems that may follow from these [67].

Most individuals with stable adolescent repNSSI, who had stopped their repNSSI in young adulthood, identified themselves as women. Even prolonged stability in repNSSI was more common among women, but not significantly so. Previous prospective studies in the present project have suggested that, for girls, repNSSI and depressive symptoms may be involved in a “vicious cycle” that might contribute to the stabilization of existing problems. For example, Lundh et al. [30] found not only that depressive symptoms at T1 were a risk factor for the development of repNSSI among girls one year later, but also that girls’ engagement in NSSI at T1 was a risk factor for increased depressive symptoms at T2. This bidirectional relationship was not found for boys. This type of mechanisms may have contributed to divergent developments of repNSSI between genders, at least in adolescence. To the extent that such bidirectional effects are at work, it is possible that they may contribute to what have been referred to in the literature as “chain reactions”, “snowball effects”, or “developmental cascades” (e.g., [68]).

A *late onset* of repNSSI among young adults who had not reported any repNSSI in adolescence was not a very common phenomenon (4.6%). Still our findings suggest that it was a real phenomenon, as it was identified in prospective data and at least not the result of a recall bias (as suggested by Gandhi et al. [7]). The pattern was neither more nor less common than expected by chance. Nor was there a gender difference in late onset, which is at odds with the findings of Gandhi et al. [7], who reported that the late onset peak was much sharper for women than for men. It should be noted, though, that our study focused on the onset of repetitive NSSI, and that some participants in our late-onset group had tested self-injurious behaviour sporadically already in adolescence.

The present study of individual developmental pathways of repNSSI also gave rise to another finding: 69.1% of the young persons in our study never reported repNSSI at any of the three time points, which means that as many as 30.9% of them *did*. This would set a quite high lower boundary for the life-time prevalence of repNSSI among the young adults in our sample that is very concerning. This should not be interpreted in terms of a life-time prevalence of clinically significant non-suicidal self-injury, however. RepNSSI was operationalized in a way that partly resembles the suggested diagnosis of NSSI disorder in DSM-5 [46], but there are crucial differences. Whereas repNSSI in the present study was defined in terms of the affirmation of at least five *instances* of NSSI during the past 6 or 12 months, the corresponding DSM-5 criterion speaks of engagement in NSSI on 5 or more *days* in the past year. For example, nothing precludes that participants in the present study who affirmed the presence of five instances of NSSI had engaged in all these NSSI behaviours on one and the same day. Furthermore, the DSM diagnosis also includes other criteria which refer to functional, motivational, and emotional aspects of NSSI. These aspects were not included in our operationalization of repNSSI. Therefore, the high lifetime prevalence of self-reported repNSSI in this study should not lead to any conclusions about the lifetime prevalence of clinically significant NSSI among young adults in the general population in Sweden. Still, the present operationalization of repNSSI may be valuable if it can contribute to new knowledge about the vicissitudes of NSSI during adolescence and onwards.

### Can different developmental repNSSI pathways be predicted?

Previous research [5, 26] has found that depression can predict onset of NSSI over shorter time periods. In a previous study of the present cohort [30] we similarly found that depressive symptoms could predict onset of adolescent repNSSI over a 1-year interval. In the present study we wanted to see if adolescent depressive symptoms could also predict late onset of repNSSI over a 10-year interval. Although the effects were of medium size at both time points (*OR*s 3.03 and 2.69, respectively), they were only nearly significant when adolescent sporadic NSSI was controlled for (T1: *p* = .062; T2: *p* = .069). Sporadic NSSI at T1 but not at T2 was a significant predictor. Based on these findings we would still suggest a continued consideration of adolescent depressive symptoms as potential risk factors of NSSI, not only in the short run but also over longer age periods. This is also consistent with the suggestion [7] that late-onset of NSSI may be a delayed manifestation of untreated adolescent psychopathology.

Moreover, poor sleep – one of the symptoms included in the depression index we used – independently predicted late onset of repNSSI even when controlling for sporadic NSSI in adolescence, although only from T2 and with a low effect size (*OR* 1.90). This finding to some extent resembles an earlier finding [31] in our research project that poor sleep among the girls was a one year-predictor of onset (new cases of repNSSI) in adolescence. This means that the finding of poor sleep as a predictor of repNSSI was made for two non-overlapping groups in our project – girls with an onset of repNSSI in adolescence and participants with a late onset of repNSSI as reported in young adulthood. The number of participants with an onset of repNSSI in young adulthood was too low, however, for replicating the regression analyses separately for each gender. Still, these findings might be reason enough to suggest that poor sleep is a marker for problematic psychological health that does not necessarily show up in the self-assessment of depressive symptoms or of other psychological difficulties. In summary, this means that the findings concerning the prediction of new cases from our one-year follow-up of the cohort in adolescence were partly replicated in the 10-year follow-up in young adulthood.

On the other hand, we could not predict the prolongation of stable adolescent repNSSI into young adulthood. Why, then, were we not able to find any predictor of the prolongation? One possible explanation is that we did not include the relevant predictors. We chose to focus on the same risk factors that had proved successful in predicting new cases of repNSSI over one year in adolescence (i.e., general psychological difficulties, depressive symptoms, and poor sleep), but it has been argued that the risk factors for maintenance are different from those for onset [8, 10]. Maybe the prolongation *versus* cessation of NSSI is to be understood primarily in terms of the *functions* of NSSI more than in terms of distal risk factors. Although the previous studies made in this area are mainly retrospective and suffer from contradictory findings, there is some evidence that intrapersonal functions of NSSI are associated with difficulties to stop engaging in NSSI [e.g., 69, 70]. To achieve more clarity in this area there is a need for more prospective studies that investigate motives for cessation that young people think about and consider before actual cessation.

Our limited success in predicting developmental patterns regarding repNSSI should also be seen in the context of the rather weak findings from other research in this area. The authors of a meta-analysis of risk factors for NSSI [8] concluded that few strong NSSI risk factors have been identified; this was so although most of the studies reviewed had considerably shorter follow-up lengths than our study. The difficulties of finding strong predictors of developmental patterns of repNSSI over a 10-year period might also be related to the period from adolescence to young adulthood being a period of much change in many areas in life simultaneously (leaving school, starting one’s work life or higher education, leaving home, finding a partner etc.). This might make it difficult to find single variables that can serve as predictors at the group level over such an extended period.

The difficulties we had to find predictors that differentiate between the two pathways with stable adolescent repNSSI but contrasting developments of repNSSI into young adulthood have parallels in previous pathway studies [20–22]. Most risk factors identified in those studies differentiated between the low NSSI trajectory class, on the one hand, and the higher NSSI trajectory classes on the other. There were some exceptions, however, of risk factors that did differentiate between different pathways involving NSSI: negative attributional style [20], family-related stress, peer victimization, and symptoms of depression and anxiety [22]. Still, the predictions in those studies cover much shorter time periods.

### What are the young adult outcomes of different repNSSI pathways?

Among the three pathway groups we chose to focus on in this study, the group with an onset of repNSSI in young adulthood appeared to fare the worst in young adulthood. This was evident both from their mean values in different psychological adjustment outcomes, and from the significant differences with the comparison group not reporting repNSSI during the 10-year period. The participants with a late repNSSI onset differed significantly from the No repNSSI pathway group in all positive and negative adjustment aspects considered, and the effect sizes were large. The Late-Onset group reported repNSSI behaviour for the first time at an age when most individuals with a stable adolescent repNSSI behaviour did not report this behaviour anymore. The social pressure against self-injurious behaviour probably is much increased in young adulthood and the threshold of an onset of this behaviour is probably much higher. In this perspective, it might be expected that it should take a more painful psychological situation for an individual to pass this threshold at that age. The repNSSI development of this group was partly predictable from symptom measures in adolescence (including sporadic NSSI at T1), however, which suggests that their poor psychological situation in young adulthood might have earlier roots.

The participants who had reported stable repNSSI only in adolescence, and had stopped their repNSSI in young adulthood, still reported significantly worse levels of stress symptoms, anxiety, and emotional dysregulation in young adulthood as compared to participants without repNSSI during the whole age period in question. These differences were large. This suggests that their overcoming the repNSSI behaviour represented a limited change in their symptom picture, leaving other impairments in their psychological adjustment. As concerns the group of participants whose repNSSI was prolonged into young adulthood, the disadvantages in comparison to the No RepNSSI group were large for most adjustment measures, but only the large difference in emotion dysregulation was significant. The low power in these comparisons constitutes a problem when it comes to evaluating the outcome in young adulthood of the prolonged repNSSI group. One possible explanation why the late onset group appeared to fare worse than this group is that the prolonged repNSSI group to some extent might have integrated the NSSI behavior into their coping strategies and/or identities in a way that the late onset group had not yet been able to do.

There were no significant differences between the three groups with different repNSSI pathways, which means that the conclusion that those with a late onset appeared to fare the worst is arguable. At the same time, the absence of significant differences cannot be interpreted as an absence of differences. The three groups were small, and the adjustment variation within the groups was rather large. Non-significant differences of medium effect sizes in the measures of positive adjustment as well as depression were found between the group with a late onset and the group with adolescence-limited stable repNSSI, which had the more positive outcome. As concerns the comparison between the adolescence-limited group and the prolongation group, however, most differences in negative adjustment were small: the participants in the adolescence-limited group did not differ much from the prolongation group in young adult symptoms of stress, anxiety, and depression.

### Strengths and limitations

Two substantial strengths of the present study were the high response rate during adolescence, and the availability of ten-year follow up data in young adulthood. At the same time, the reduced response rate at the ten-year follow up represents an important limitation. This response rate, however, is comparable to the response rate in other similar studies (e.g., [71, 72]).

Another limitation is that we only have three points of measurement, and that there is a 9-year time gap in our longitudinal sequence of data collections (that is, between T2 and T3), unfortunately during an important transition period in the lives of the participants. Additionally, the DSHI-9r asks about the presence of NSSI only during the past 6 months (at T1 and T2) or 12 months (at T3), whereas the time intervals between T1 and T2, and between T2 and T3, were longer than these periods of report; this means that we do not have any data on NSSI during 6 months after T1, and during an 8-year period after T2. It also means that the repNSSI assessments are not exactly comparable.

We merely used self-report measures of psychological difficulties in adolescence and young adulthood. Reports from parents, teachers, friends, and partners might have contributed with other perspectives on psychological difficulties among the participants. Further, the Strengths and Difficulties Questionnaire we used in the adolescent data collections may be questioned as to its ability to capture severe emotional and interpersonal problems in adolescence, because it does not include any questions about depression, shame, or self-hatred. And although the specially constructed Depression Index covered most of the criteria of major depression, as defined by the DSM-IV [53], it did not cover the criteria of weight loss or weight gain, nor recurrent thoughts of death and suicide. This means that the depression index used in the present study does not do full justice to the psychiatric notion of major depression.

Finally, it should be noted that gender findings are limited by the use of only a binary conceptualization of gender. At T3 we did include the option to respond other than man or woman, but no one in the present longitudinal sample chose that alternative. It also remains to be seen to which extent the present findings are generalizable to other communities and cultures.

## Conclusions

There are three main findings of the present study. First, the individual developmental pathway of stable repNSSI across all three time points over the 10-year period showed considerable stability, which is congruent with conceptualizations of NSSI as self-reinforcing in nature. But the results also indicate that most adolescents who engage in stable repNSSI during adolescence stop engaging in it before reaching young adulthood.

Second, however, these seemingly hopeful results are tempered by the finding that those who stopped engaging in repNSSI still had worse negative psychological adjustment in young adulthood than those who had never engaged in repNSSI. There was not any clear evidence of differences in young adult adjustment between this group and the group who continued to engage in repNSSI in young adulthood; nor could these two groups be differentiated by any adolescent risk factors.

Third, the results indicate that late onset of repNSSI as reported in young adulthood may be predictable from symptom measures in adolescence. Although these findings are less robust (e.g., poor sleep was a significant predictor only at T2, sporadic NSSI was a significant predictor only at T1, and depression was only a nearly significant predictor at T1 and T2), this suggests that psychological symptoms among adolescents who do not engage in repNSSI at the time may still be a risk factor for an onset of repNSSI many years later. Altogether these findings point to the need for a broad developmental approach to the study of young people’s psychological health if we are to come to grips with repNSSI.

## Data Availability

The datasets analysed during the current study are not publicly available due to ethical restrictions. The datasets are available from the corresponding author on reasonable request and after approval from the Swedish Ethical Review Authority.

## Appendix A

### First-order configural frequency analysis

In the present study, the participants reported NSSI at three different time points: when they were in grades 7 and 8 (T1), when they were in grades 8 and 9 (T2), and at a follow-up after 10 years (T3). At each time point, the measure of NSSI was dichotomized into presence/ absence of repetitive NSSI (repNSSI). Reports of at least 5 instances of self-harm during the past six (T1 and T2) or twelve (T3) months rendered a repNSSI value of 1; and 0-4 instances of self-harm rendered a value of 0. With dichotomous measures at three time points, there are eight (2 × 2 × 2) theoretically possible value patterns or *configurations*.

The frequencies of these value patterns were analysed with *Configural Frequency Analysis* (CFA; [73]) in which the observed frequencies of all value patterns are compared to the frequencies *expected by chance* from a *base model*. We used a *first-order CFA*, in which the base model is a model of *independence*, in which differences in marginal frequencies are taken into account [19].

In practice, the analysis was performed as follows:

1. The dichotomous measures of repNSSI at the three time-points were considered as three independent time-specific variables: *RepNSSI at T1*, *RepNSSI at T2*, and *RepNSSI at T3*.
2. The three variables were cross-tabulated, resulting in a three-way contingency table with 2 × 2 × 2 = 8 cells. Each cell corresponded to a specific value pattern, in turn signifying a specific developmental pathway of repNSSI.
3. The expected frequencies were computed based on the model of *independence* and the *marginal frequencies* of the cross-tabulation (cf. the computation of expected frequencies in a *χ*^2^-analysis of independence in a two-way contingency table). This means, that the differences in frequency of repNSSI between the three time points were included in the computations of the expected frequencies.
4. The computations of expected frequencies for the eight configurations in our case, that is, the eight developmental value patterns, are presented in Table A1.
5. The two-tailed probability of the observed frequency of the specific value pattern under the model of independence was given by the binomial distribution [74].

Developmental patterns that are observed significantly *more* often than would be expected by chance are called *types*. Developmental patterns that are observed significantly *less* often than expected by chance are called *antitypes*.

The choice of base model has consequences for what patterns that may turn up as types and antitypes and therefore, the choice should be made according to what you are interested in discovering [19]. With zero-order CFA, for example, the base model proposes a uniform distribution of cases in the cross-tabulation, and significant discrepancies from the uniform distribution that are caused by age differences in repNSSI will turn up as types, in addition to discrepancies caused by associations between age levels.

We chose a first-order CFA with an independence model as the base model, because we wanted to identify all types and antitypes that appeared because of associations between time points, while we considered the repNSSI frequency at each time point as given. As described at Step 3, the differences in how common repNSSI was at different age levels were included in the computations of expected frequencies and would not give rise to types and antitypes. It is important to note, however, that with this base model, pairwise associations between time points that were independent of the third time point could give rise to types and antitypes. If we only had been interested in three-way associations (that is, value patterns that involve dependence between all three time points) we would have had to use a second-order CFA in which pairwise associations were included in the base model (see [19]).

The CFA was carried out using the computer program ROPstat [64].

**Table A1:** Value Patterns and Computations of Expected Frequencies Based on an Independence Model of Absence or Presence of RepNSSI at Three Age Levels. The Computations Were Made from Marginal Frequencies in a Three-Way Contingency Table (N = 475)

		RepNSSI at T3	
RepNSSI at T1	RepNSSI at T2	0 (n = 428)	1 (n = 47)
0 (n = 400)	0 (n = 372)	Value pattern: 000 $\frac{400}{475}\times \frac{372}{475}\times 428$	Value pattern: 001 $\frac{400}{475}\times \frac{372}{475}\times 47$
	1 (n = 103)	Value pattern: 010 $\frac{400}{475}\times \frac{103}{475}\times 428$	Value pattern: 011 $\frac{400}{475}\times \frac{103}{475}\times 47$
1 (n = 75)	0 (n = 372)	Value pattern: 100 $\frac{75}{475}\times \frac{372}{475}\times 428$	Value pattern: 101 $\frac{75}{475}\times \frac{372}{475}\times 47$
	1 (n = 103)	Value pattern: 110 $\frac{75}{475}\times \frac{103}{475}\times 428$	Value pattern: 111 $\frac{75}{475}\times \frac{103}{475}\times 47$

*Note*. 0 = no repNSSI, 1 = repNSSI.

## Footnotes

1 *Pathway* is the concept we use here for the individual developmental course of NSSI. At the methodological level, we have used the term *pattern* to designate the observed individual NSSI data series over time. *Trajectory* is the methodological term used in research studies that aim to identify *latent* trajectories underlying/explaining the individual observed data patterns.

2 We also made an alternative imputation of our data with the Expectation-Maximization algorithm, but the average NSSI scores at T1 and T2 were only very slightly higher with this imputation and it did not change the categorization of any of the participants.

## Abbreviations

CFA: Configural frequency analysis
NSSI: Non-suicidal self-injury
repNSSI: Repetitive non-suicidal self-injury
